# The Effects of Endoplasmic-Reticulum-Resident Selenoproteins in a Nonalcoholic Fatty Liver Disease Pig Model Induced by a High-Fat Diet

**DOI:** 10.3390/nu12030692

**Published:** 2020-03-04

**Authors:** Pengzu Wang, Zhuang Lu, Meng He, Baoming Shi, Xingen Lei, Anshan Shan

**Affiliations:** 1 Institute of Animal Nutrition, Northeast Agricultural University, Harbin 150030, China; princewpz@163.com (P.W.); luzhuang0416@163.com (Z.L.); hemeng0210@163.com (M.H.); shibaoming1974@163.com (B.S.); 2 Department of Animal Science, Cornell University, Ithaca, NY 14853, USA; xl20@cornell.edu

**Keywords:** Selenium, high-fat diet, liver, oxidative stress, apoptosis

## Abstract

The present study aimed to investigate the intervention of selenium in the oxidative stress and apoptosis of pig livers, which were induced by a high-fat diet, and the effects of four endoplasmic reticulum (ER)-resident selenoproteins in the process. A 2 × 4 design trial was conducted that included two dietary fat levels (BD = basal diet and HFD = high-fat diet) and four dietary Se supplementation levels (0, 0.3, 1.0, and 3.0 mg/kg of the diet, in the form of sodium selenite (Na_2_SeO_3_)). Our results indicated that the HFD significantly increased the activities of alanine aminotransferase (ALT) and aspartate aminotransferase (AST) in the serum, as well as the degree of steatosis, the content of malondialdehyde (MDA), the apoptotic rate, and the level of mRNA caspase-3 in the liver compared to their BD counterparts (*p* < 0.05). Moreover, these parameters in the HFD groups were more significantly reduced (*p* < 0.05) for a Se concentration of 1.0 mg/kg than for the other concentrations. Further, for both the BD and HFD, the groups supplemented with 1.0 mg/kg Se showed the highest mRNA level of selenoprotein S. In conclusion, the consumption of an HFD can induce oxidative damage and apoptosis in the liver. This shows that the supplementation of Se at 1.0 mg/kg may be the optimum concentration against damage induced by HFD, and Sels may play a key role in this process.

## 1. Introduction

In the last two or three decades, nonalcoholic fatty liver disease (NAFLD) has become increasingly prevalent in many countries worldwide, as people continue to eat excessive dietary fat [1]. The pathological processes of NAFLD start with a fatty liver only (steatosis) and transition to nonalcoholic steatohepatitis (NASH) and, ultimately, cirrhosis [2]. A “two-hit” NASH pathogenesis model was proposed by Day (1998), who considered fat accumulation as the first hit and increased oxidative stress as the significant second hit [3]. The chronic intake of a high-fat diet (HFD) can abnormally increase hepatic triglyceride and cholesterol levels, thus potentially promoting lipid peroxidation and oxidative stress within hepatocytes [4]. Chronic oxidative stress that is mainly induced by mitochondrial dysfunction can develop into NAFLD and metabolic syndrome [5]. Moreover, oxidative stress can intervene in the functional expression of the proapoptotic signal protein genes of Bax (Bcl2-associated X protein), caspase-3, and p53 (tumor protein 53), as well as the prosurvival signal protein gene Bcl2 (B-cell lymphoma 2), which may ultimately induce apoptosis [6,7,8]. Emerging data have shown that patients with NASH have a significant hepatocellular apoptosis rate that correlates with disease severity [9,10]. This indicates that the etiology of NASH could be caused by apoptosis, and antiapoptotic therapy may be the cure for this syndrome. 

Selenium (Se), an essential trace element that shows antioxidant active oxygen free radical scavenging, defends the organs and tissues against oxidative damage and improves the body’s immune system [11]. However, Se can also induce glutathione metabolism and oxidative stress both in nature and in laboratory studies [12]. When dietary Se levels increase, hepatic glutathione peroxidase (GPx) activities occur dose-dependently to prevent oxidative glutathione peroxidase (GSSG), ultimately causing an increase in hepatic lipid peroxidation [13]. Selenium deficiency can lead to muscular dystrophy, endemic fatal cardiomyopathy (Keshan disease), and chronic degenerative diseases in humans. If patients were to use selenium supplementation alone or in combination, this could be prevented [14]. The metabolic roles of Se achieve Se’s biological function through its incorporation into selenoproteins [15], which contain a sole amino acid, selenocysteine (Sec) [16]. At present, 25 human selenoproteins have been identified [17], and many of them are antioxidant enzymes. Researchers have found at least four selenoproteins, including selenoproteins N, T, S, and K (SEPN1, SELT, SELS, and SELK), in the endoplasmic reticulum (ER). Sepn1, Selt, Sels, and Selk, respectively, encode these novel proteins [18,19,20,21]. Since these proteins play essential roles in intracellular calcium concentration regulation and ER stress [22,23], they may influence the pathological processes of NASH induced by HFD. Moreover, Se has been considered to have insulin-mimetic and antidiabetic properties for a long period of time [24,25], but more recent epidemiological studies have suggested that high plasma Se and selenoproteins P (SelP) levels are associated with insulin resistance and NAFLD [26,27]. However, we still know little about whether ER-resident selenoproteins affect the pathological processes of NASH.

The animal models for NASH are considered to be a useful tool to investigate the potential mechanisms involved in its pathogenesis. Recently, researchers using HFD have developed several nutritional animal models for NASH, but these models were concentrated mainly on rodent animals [28,29]. Compared to rodents, pigs share a greater metabolic similarity and disease susceptibility with humans in developing type 2 diabetes or metabolic syndrome, a similarity that can be exploited in human nutrition and medicine studies [30]. In the current study, we used HFD-fed pigs as a model of NASH to investigate the effects of ER-resident selenoproteins on the pathological processes of NASH. The biological superiority of HFD-induced NASH in this model is more appropriate as the pigs consumed their diets ad libitum, which is more similar to human eating patterns, rather than being force-fed. The levels of hepatic lipids, antioxidant status, and the function of the liver were examined. TdT-mediated dUTP nick-end labeling (TUNEL) was adopted to test DNA fragmentation as an indicator of cell apoptosis. Moreover, the liver’s histopathology and ultrastructure were observed. The mRNA expression of the ER-resident selenoproteins and apoptosis-related proteins was measured. The objective of our study was to investigate the intervention of selenium in the high-fat-diet-induced oxidative stress and apoptosis of pig livers and the effects of four endoplasmic reticulum (ER)-resident selenoproteins in this process.

## 2. Methods

### 2.1. Treatment of Experimental Animals

Forty healthy, uncastrated boars (Duroc × Landrace × Yorkshire), 40 days of age, with an initial body weight (BW) of 10 ± 0.72 kg, were chosen and randomly allotted to treatments in a 2 × 4 factorial arrangement. The treatments included two levels of dietary fat and four levels of Se. The pigs were fed a Se-deficient, corn–soybean type meal (produced in the Se-deficient region of Heilongjiang, China) basal diet (BD) or a high-fat diet (HFD) (Table 1). The HFD was supplemented with fish oil at 1% and lard at 3% (10–30 kg), 7% (30–60 kg), or 11% (>60 kg). The basal concentration of Se in the BD and HFD without additional Se was <0.03 in all periods. Se was added at 0, 0.3, 1.0, or 3.0 mg/kg in the form of sodium selenite (Na_2_SeO_3_) (Sigma-Aldrich, St. Louis, MO, USA) in the BD and HFD. All pigs were placed in individual cages with fully slatted floors, a single-hole feeder, and a nipple waterer, with feed and water provided ad libitum. The temperature of the animal-housing facility was maintained at 20–25 °C. The trial was conducted at the Animal Experimental Center of Northeast Agricultural University, Harbin, China. The experimental period lasted for 16 weeks. All pigs were deprived of food for 12 h and sacrificed through an injection containing a combination of ketamine (20 mg/kg BW; Fujian Gutian Pharma Co. Ltd.) and thiamylal (10 mg/kg BW; MedchemExpress, Shanghai) at the end of the study. The livers were sampled, rinsed with ice-cold sterile deionized water, immediately frozen in liquid nitrogen, and then stored at −80 °C until analysis. Concurrently, blood samples were collected (5 mL) via the anterior vena cava with an anticoagulant free vacuum tube from individual pigs and centrifuged at 3000× *g* for 15 min at 4 °C to obtain the serum; they were then stored at −80 °C for measurements. All experimental procedures were approved by the Animal Ethics Committee of the Northeast Agricultural University (NEAU-2011-9) and were performed according to the guidelines for animal experiments of the National Research Council Guide.

### 2.2. Growth Performance

The pigs’ body weights and feed intake values were recorded from the beginning of the study to the end to calculate the average daily gain (ADG) and average daily feed intake (ADFI). The carcass weights and lengths were measured at the end of the study.

### 2.3. Serum Enzyme Activity Assays 

We assayed the activities of serum alanine aminotransferase (ALT) and aspartate aminotransferase (AST) using the method of Reitman and Frankel (1957) [31].

### 2.4. Determination of Liver Biochemical Indexes

The liver tissues (1 g) and 9 mL of ice-cold phosphate buffer saline were homogenized to prepare the liver homogenate. Then, the homogenates were centrifuged at 1000 rpm for 15 min at 4 °C to discard any cell debris, and the supernatant was prepared for the measurement of total cholesterol (TC), triglycerides (TG), malondialdehyde (MDA), and glutathione peroxidase (GPx). The corresponding diagnostic kits (Nanjing Jiancheng Bioengineering Company, Nanjing, China) were used, following the instructions of the manufacturers. Total protein contents were measured using the method of Lowry et al. (1951) [32]. Tests were performed using a UV-2401 spectrophotometer (Shimadzu Corporation, Tokyo, Japan).

### 2.5. Histological Examinations 

After the removal from each animal, two small portions of liver were quickly saturated in 10% neutral-buffered formalin solution for at least 24 h. The fixed specimens were dehydrated through a graded series of ethanol, cleared in xylene, and embedded in paraffin. Sections 5 μm thick were obtained and stained with hematoxylin and eosin for examination under light microscopy (XDS-1B, Olympus, Japan) using the prepared paraffin blocks.

### 2.6. Ultrastructural Observation 

For the ultrastructural examination, the liver tissues were dissected to 1 mm^3^ and immediately fixed in 2.5% glutaraldehyde phosphate buffer saline (*v*/*v*, pH 7.2), postfixed in 1% osmium tetroxide (*v*/*v*), and the specimens were stained with 4.8% uranyl acetate. Then, the fixed specimens were dehydrated in a graded series of ethanol and embedded in Araldite. Ultrathin (less than or equal to 90 nm) sections were sliced, mounted on coated copper grids, washed in propylene oxide, and impregnated with epoxy resins; then, the sections were poststained with uranyl acetate and lead citrate. The specimens were observed using a transmission electron microscope (GEM-1200ES, Japan).

### 2.7. Gene Expression by Real-Time PCR

Total mRNA was isolated from the liver samples using a TRIzol reagent following the instructions of the manufacturer (Invitrogen, Carlsbad, CA, USA). A spectrophotometer measured the RNA concentration at 260/280 nm. To estimate the quality of the RNA, we detected the number of bands by agarose gel electrophoresis. RNA was reverse transcribed into cDNA with a cDNA reverse transcription kit (Takara, Dalian, China) and a thermal cycler following the manufacturer’s instructions. The RT reaction was conducted in 20 μL of the reaction mixture at 37 °C for 15 min and terminated by heating the mixture at 85 °C for 5 s followed by cooling at 4 °C.

The primers for the Bax, Bcl-2, caspase-3, p53, and four ER-resident selenoproteins (SEPN1, SELT, SELS, and SELK) were designed with the software Primer Premier 5.0 based on known pig sequences in Genbank and listed in Table 2. The primers’ specificities were tested by a BLAST analysis of the NCBI database. Meanwhile, β-actin was amplified as a housekeeping gene. Quantitative real-time PCR was performed on an ABI PRISM 7500 Detection System (Applied Biosystems, Foster City, CA, USA). Reactions were performed with 2.0 μL of the first-strand cDNA and 0.8 μL of the sense and antisense primers in a final volume of 20 μL, as recommended by the SYBR real-time PCR kit (Takara, Dalian, China). The RT-PCR conditions were as follows: 1 cycle at 95 °C for 30 s, 40 cycles at 95 °C for 5 s, and 60 °C for 34 s. All of the PCR reactions were performed in triplicate. The results were normalized by β-actin gene expression (the β-actin expression in the samples was consistent). The relative mRNA expression was calculated using the 2^−ΔΔCt^ method [33].

### 2.8. Measurement of Apoptosis 

Apoptosis was tested using the terminal dUTP nick-end labeling (TUNEL) method. The TUNEL test was performed using a cell apoptosis detection kit (Roche) following the manufacturer’s instructions. In brief, paraffin sections of the liver were deparaffinized in two new changes of xylene for 10 min each. The sections were gradually hydrated with gradient ethanol and finally introduced to water. Deparaffinized tissue sections were treated with proteinase K, and the endogenous peroxidase activity was blocked by incubation with fresh 3% H_2_O_2_ for 30 min. The sections were incubated at 37 °C with Tdt (terminal deoxynucleotidyl transferase)/nucleotide mixture for 1 h. Then, the slides were rinsed with PBS for 10 min at room temperature after the reaction was stopped. The tissue sections were incubated with the anti-digoxigenin-peroxidase antibody at room temperature for 30 min. The positive cells were detected using a diaminobenzidine (DAB) solution. The number of apoptotic cells on each slide was recorded from at least five different fields using the Image-Pro Plus software (version 6.0 for Windows) with the aid of a microscope (BA400, Motic) at 400× magnification.

### 2.9. Statistical Analysis

The data were analyzed using SPSS for Windows (version 19; SPSS). The main effects of the fat and Se concentrations were analyzed as a 2 × 4 factorial arrangement of treatments using a two-way ANOVA. The mean comparisons were conducted using a Bonferroni post hoc test, while the main effect was significant. If an interaction between dietary fat and Se was found, the means were conditionally compared. At the same Se concentrations, the effects of the fat level were tested with a one-way ANOVA. The data are expressed as the means ± SDs, and *p* < 0.05 was considered significant.

## 3. Results

### 3.1. Growth Performance

The growth performance data for the Se and fat dietary treatments during the study are shown in Table 3. There were no SeΧfat interactions, except for carcass length (*p* < 0.01). Se dosage had no significant effects on growth performance (*p* > 0.05). In contrast, fat levels affected growth performance (*p* > 0.01). At the end of the trial, the ADG values of the HFD groups were higher than those of the BD groups at the same Se dosage (*p* < 0.05).The HFD groups also gained more body weight than the BD groups at the same Se dosage (*p* < 0.05). The HFD groups took in more feed than the BD groups (*p* < 0.05), except at a Se dosage of 1.0 mg/kg.

### 3.2. Serum ALT and AST Analysis

Table 4 shows that the activities of ALT and AST in the HFD groups were significantly elevated (*p* < 0.05) at various Se concentrations compared to their BD counterparts. In the HFD groups, the activities of ALT and AST were significantly lower (*p* < 0.05) for a Se concentration of 1.0 mg/kg than for other concentrations. The activities of ALT in the BD groups were not significantly different among the treatment groups. 

### 3.3. Antioxidant Enzyme Activity

According to the results in Table 5, the activities of Gpx in the HFD groups were significantly reduced (*p* < 0.05) at Se dosages of 0.3, 1.0, and 3.0 mg/kg compared to their BD counterparts. The MDA contents in the HFD groups were elevated significantly (*p* < 0.05) at various Se concentrations compared to their BD counterparts. In the HFD groups, the group supplemented with 1.0 mg/kg Se showed the highest activities of Gpx and the lowest contents of MDA.

### 3.4. Concentrations of TC and TG

As shown in Table 6, the concentrations of TC and TG in the HFD groups were significantly elevated (*p* < 0.01) at various Se concentrations compared to their BD counterparts. In the HFD groups, the concentrations of TC and TG were more significantly reduced (*p* < 0.05) for a Se concentration of 1.0 mg/kg than for other concentrations. The concentrations of TC and TG in the BD groups were significantly elevated (*p* < 0.05) for a Se concentration of 3.0 mg/kg. 

### 3.5. Histological Analysis

**Figure 1:**
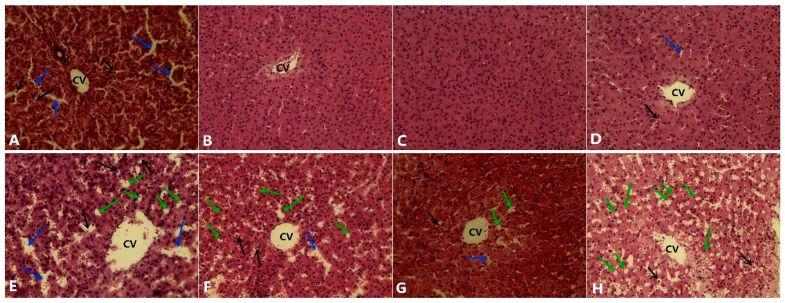
Hematoxylin and eosin-stained liver slices from the treated pig. (**A**) Pigs fed a normal diet with 0 mg/kg Se; (**B**) pigs fed a normal diet with 0.3 mg/kg Se; (**C**) pigs fed a normal diet with 1.0 mg/kg Se; (**D**) pigs fed a normal diet with 3.0 mg/kg Se; (**E**) pigs fed a high-fat diet with 0 mg/kg Se; (**F**) pigs fed a high-fat diet with 0.3 mg/kg Se; (**G**) pigs fed a high-fat diet with 1.0 mg/kg Se; (**H**) pigs fed a high-fat diet with 3.0 mg/kg Se. The blue arrow indicates the sinusoids enlarged between the plates of the hepatocytes. The black arrows indicate hepatic cell necrosis. The green arrow indicates a micro- and macrovesicular fatty change. CV: central vein. (H.E. × 400).

Histological examination showed that the structure of the hepatic tissue was normal in the BD groups for Se concentrations of 0.3 and 1.0 mg/kg (Figure 1B,C). The livers of pigs treated with 0 and 3.0 mg/kg presented mild liver damage, intrahepatic hemorrhaging, and a destroyed liver structure (Figure 1A,D). Pigs fed the HFD developed a high degree of steatosis at a Se concentration of 0 mg/kg, and the micro- and macrovesicular fatty changes were distinct (Figure 1E) in the liver. However, the damage was partially mitigated in the HFD groups treated with Se concentrations of 0.3 and 1.0 mg/kg (Figure 1F,G). The high-fat diet supplemented with 3 mg/kg Se demonstrated a relatively higher hepatic lipid accumulation compared to the groups treated with 0.3 and 1.0 mg/kg.

### 3.6. Ultrastructural Alterations

**Figure 2:**
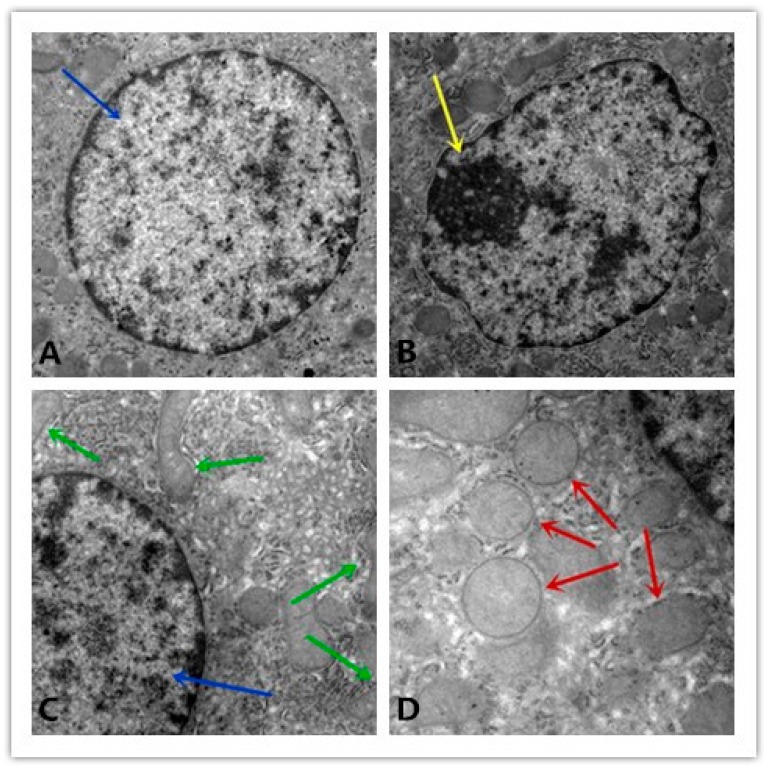
Liver ultrastructure observations. (**A**) Normal nucleus; (**B**) apoptosis nucleus; (**C**) normal mitochondria; (**D**) swelled mitochondria. The blue arrow shows a normal nucleus that contains equally distributed chromatin, the yellow arrow shows chromatin aggregation in the cell nucleus, the green arrow shows normal mitochondria, and the red arrow shows mitochondria swelling with the lysis of the cristae. Magnifications: 15,000×.

The normal pig liver cells showed structural integrity, with evenly distributed chromatin (Figure 2A) and rich cristae in the mitochondria (Figure 2C). The apoptosis cells of the liver indicated unclear membrane and chromatin aggregation (Figure 2B). The mitochondria of the apoptosis liver cells swelled with the lysis of the cristae and changed into a vacuole (Figure 2D).

### 3.7. p53, Bax, Bcl-2, Caspase-3 Level, and Apoptosis

**Figure 3:**
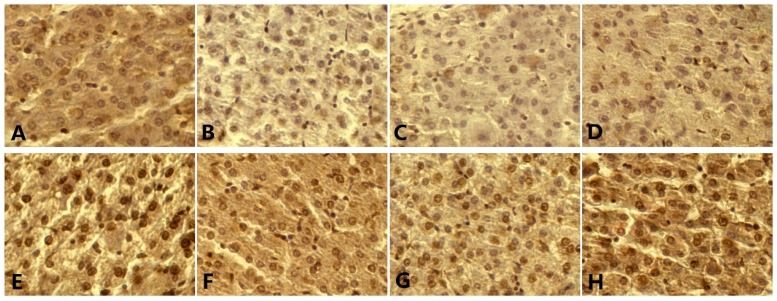
Apoptotic nuclei labeled (brown) with the TUNEL method. (**A**) Pigs fed a normal diet with 0 mg/kg Se; (**B**) pigs fed a normal diet with 0.3 mg/kg Se; (**C**) pigs fed a normal diet with 1.0 mg/kg Se; (**D**) pigs fed a normal diet with 3.0 mg/kg Se; (**E**) pigs fed a high-fat diet with 0 mg/kg Se; (**F**) pigs fed a high-fat diet with 0.3 mg/kg Se; (**G**) pigs fed a high-fat diet with 1.0 mg/kg Se; (**H**) pigs fed a high-fat diet with 3.0 mg/kg Se. Original magnification, ×400.

**Figure 4:**
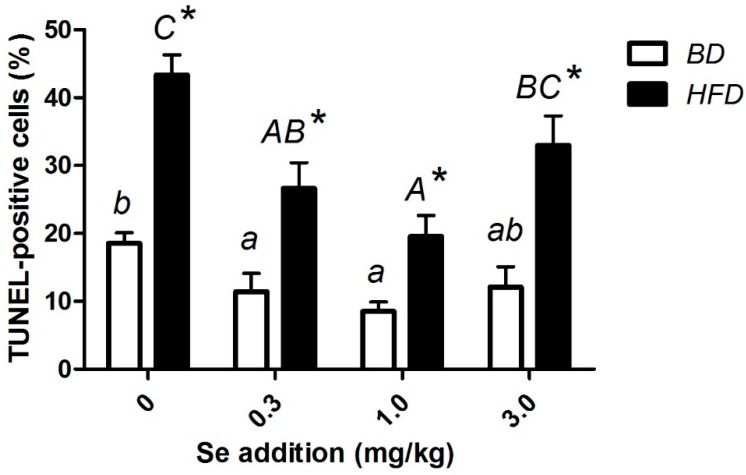
Levels of cell apoptosis of the liver determined by the TUNEL assay. * Different from the BD at that Se addition, *p* < 0.05. ^a–b^ Within BD groups, means without a common letter differ, *p* < 0.05. ^A–C^ Within HFD groups, means without a common letter differ, *p* < 0.05.

**Figure 5:**
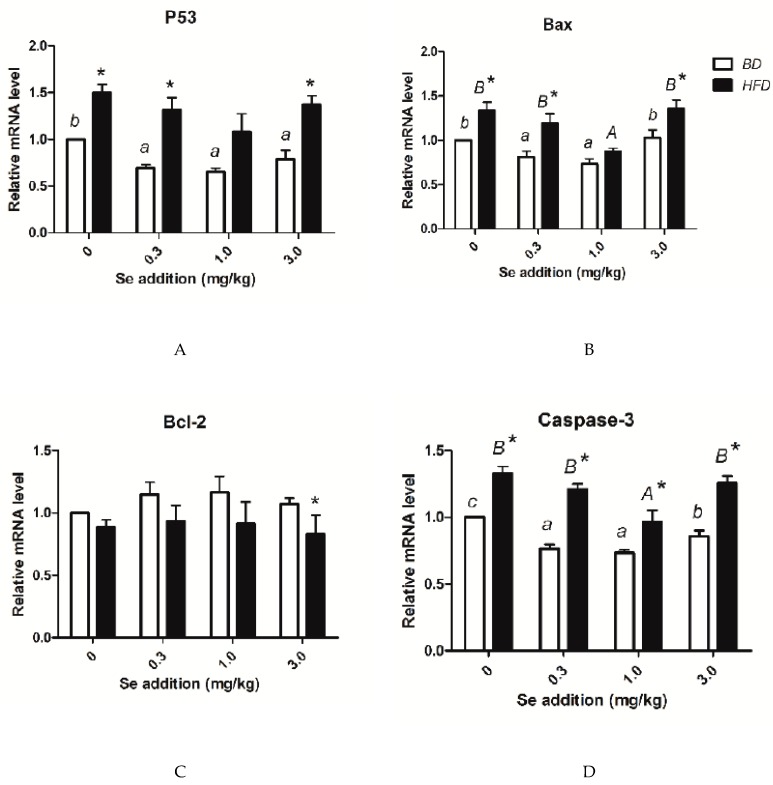
Levels of apoptosis gene mRNA. (**A**) The mRNA level of P53; (**B**) the mRNA level of Bax; (**C**) the mRNA level of Bcl-2; (**D**) the mRNA level of Caspase-3. * Different from the BD at that Se addition, *p* < 0.05. ^a–c^ Within BD groups, means without a common letter differ, *p* < 0.05. ^A–B^ Within HFD groups, means without a common letter differ, *p* < 0.05.

The pictures of the TUNEL method show the typical morphological and biochemical features of apoptosis. Chromatin condensed and cells shrank, while membrane-bound cell and DNA fragments joined together (Figure 3). The apoptotic rate and the mRNA level of caspase-3 were significantly elevated (*p* < 0.05) at various Se concentrations compared to their BD counterparts. For both the BD and HFD groups, the highest ratio of apoptotic cells was observed at a Se concentration of 0 mg/kg, and the lowest ratio of apoptotic cells was observed at a Se concentration of 1.0 mg/kg (Figure 4). As the Se concentration increased, the expressions of p53, Bax, and caspase-3, and the percentage of apoptotic cells first decreased and were minimized at a Se concentration of 1.0 mg/kg in the BD and HFD groups and then increased (Figure 5).

### 3.8. Relative mRNA Amounts of Selenoprotein Genes

**Figure 6:**
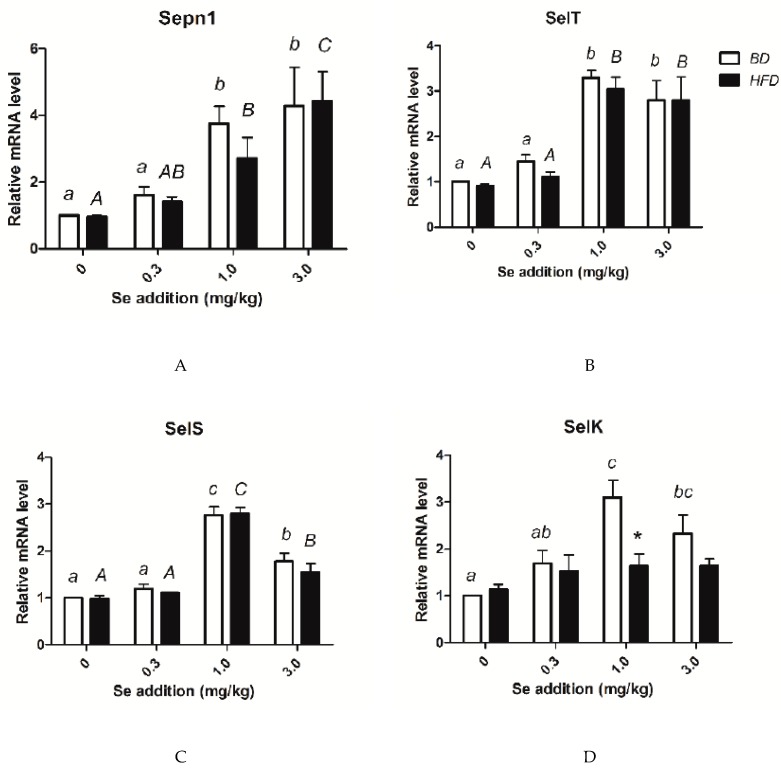
Levels of selenoprotein gene mRNA. (**A**) mRNA levels of Sepn1; (**B**) mRNA level of Selt; (**C**) mRNA level of Sels; (**D**) mRNA level of Selk. * Different from the BD at that Se addition, *p* < 0.05. ^a–c^ Within BD groups, means without a common letter differ, *p* < 0.05. ^A–C^ Within HFD groups, means without a common letter differ, *p* < 0.05.

As shown in Figure 6, the expression of Sepn1, Selk, Selt, and Sels showed no differences between the HFD and BD groups at the same Se concentration, except for Selk in the HFD 1.0 mg /kg Se group, which presented a significantly higher expression than the expression in the BD 1.0 mg /kg Se group. In both the HFD and BD groups, the expression of Sepn1 increased with the dietary Se level. The expression of Selt in the 1.0 and 3.0 mg/kg Se groups was significantly higher than the expressions of the 0 mg/kg group and the 0.3 mg/kg groups (both BD and HFD). For both the BD and HFD, the groups supplemented with 1.0 mg/kg Se showed the highest mRNA level of Sels. In the BD groups, the 1.0 mg Se/kg group showed the highest expression of Selk. There were no significant differences in the expression of Selk for the various Se concentration groups fed the HFD.

## 4. Discussion

The recommended dietary allowance of selenium for adults is 55 μg/day per person in the USA [34]. According to data from the 2009–2010 National Health and Nutrition Examination Survey (NHANES), the average daily Se intake of adult men is higher (134 μg from foods and 151 μg from foods and supplements) than that of adult women (93 μg from foods and 108 μg from foods and supplements) in the USA [35]. This value is double or triple the recommendation. Some researchers have studied the relationship between high selenium intake and T2DM, cancer, or other metabolic diseases by giving their subjects 200–600 μg selenium [36]. Pigs share a more significant metabolic similarity and disease susceptibility with humans, but there are still some differences. Pigs, for example, grow faster (from birth weight to 220 kg within less than 6 months) than humans. Thus, they need more nutrients than humans. The recommended dietary selenium for growing pigs is 0.3 mg/kg in the diet by National Research Council (NRC). Thus, we chose 1.0 mg/kg, nearly triple the recommendation.

Nonalcoholic fatty liver disease (NAFLD), which is often complex, with critical complications like obesity and/or insulin resistance, has become the most frequent complication of chronic liver disease in the world [37]. NAFLD develops clinical symptoms when the metabolites of the overtaken lipids accumulate in the liver [38]. In this trial, we succeeded in establishing a pig model with NAFLD via HFD. The hepatic pathology tests revealed a simple fatty liver, even steatohepatitis, with characteristic pathological changes.

At the same time, the HFD groups developed lipid metabolism disorders accompanied by liver lipid accumulation (higher TC and TG concentrations), hepatic function damage (higher serum ALT and AST levels), oxidative stress, and the resulting lipid peroxidation (a reduction of Gpx activity and increase of MDA content), which resembles the clinical symptoms of human NAFLD. Consequently, this pig model could be used to explore the pathogenesis of NAFLD. In the HFD groups, the pigs in the group with a Se concentration of 1.0 mg/kg showed the greatest alleviation of lipotoxicity. In both the BD and HFD groups, the pigs treated with 0 mg/kg Se exhibited more severe injury than the pigs with 0.3 and 1 mg/kg. This suggests that decreases in the liver’s Se reserves led to a decline in peroxide scavenging and hepatocyte membrane lipid oxidative damage, causing hepatocyte necrosis and variability, and ultimately developing a vicious circle [39].

In contrast, the fat deposition and damage of the liver in the HFD groups were more severe at the Se dosage of 3.0 mg/kg than at the Se dosages of 0.3 mg/kg and 1.0 mg/kg. A more recent epidemiological study suggested that super-nutritional selenium intake may be a cause of potential insulin resistance (IR) [40,41]. IR may enhance hepatic fat accumulation and oxidative damage by increasing free fatty acid delivery and the effect of hyperinsulinemia, which stimulates anabolic processes [42]. This may explain why a high dosage of selenium and fat damaged the liver.

Emerging data have shown that the hepatocellular apoptosis of patients with NASH is significantly increased and correlates with disease severity [9]. This indicates that the etiology of NASH could be caused by apoptosis, and this syndrome could be treated by antiapoptotic therapy. The present study reveals a high ratio of hepatocyte apoptosis in a NASH pig model induced by HFD. This finding is in agreement with the conclusions from both past human studies of NASH patients [9,10] and animal models of NASH induced by HFD [28,43]. Increasingly more evidence indicates that NASH may be a mitochondrial disease. When NASH is developing, the electron transport chain impairs oxidative phosphorylation, as hepatic tissues are highly dependent on oxidative metabolism and, therefore, highly vulnerable to mitochondrial impairment. The results of these ultrastructural alterations indicate that the mitochondria in apoptotic hepatocytes swell with the lysis of the cristae and change into a vacuole. This result suggests that the mitochondrial pathway is involved in the process of apoptosis. The trial revealed that, in all stages of liver steatosis, the proapoptotic proteins p53 and Bax are increasingly expressed in hepatocytes, and the antagonistic protein Bcl-2 is diminished, especially in normal hepatocytes. However, the expression of antagonistic protein Bcl-2 is slightly decreased when steatosis is intensified. Bcl-2 seems to play a minor role in the process of steatosis. In the livers of various murine NAFLD models, p53 was found to be upregulated [44]. Hepatocyte apoptosis is associated with p53 activation in mice fed HFD [45]. Also, the level of steatosis and p53 expression in the human liver are positively correlated [46]. According to all these findings, p53 activation may be a broader metabolic event that is significantly involved in the pathogenesis of a fatty liver, which not only facilitates apoptosis and oxidative stress but also generates hepatic abnormalities such as insulin resistance and steatosis. The mechanisms of the underlying apoptosis stimulated by p53 expression in hepatocytes have been observed in mice under laboratory conditions [47,48].

p53 might directly alter the transcriptional activity of genes associated with cell death and then initiate apoptosis [49,50]. p53 decreased Bcl-2 expression and increased Bax expression [51]. Under normal conditions, Bax can be found in the cytosol or loosely attached to the membranes in a monomeric form. After activation, Bax, which is cross-linkable as a homodimer, moves into the mitochondria and becomes an integral membrane protein. Bax may form channels that allow for the release of apoptosis-related proteins, such as cytochrome c, from the mitochondria to propagate the apoptotic pathway after insertion into the membranes [52,53]. Conversely, Bcl-2 has an inhibitory role in the translocation of cytochrome c and prevents the activation of cytosolic caspases and apoptosis [54,55]. Therefore, a Bax:Bcl-2 ratio might be necessary for the mitochondrial-dependent apoptosis cascade for the release of cytochrome c. These findings suggest that, although the Bcl-2 mRNA level does not change, an increase in the Bax mRNA level might play a critical role in the hepatocyte apoptosis pathway. Caspases play an important role in the execution of apoptosis in several cell types [56]. Caspase-3 appears to be an essential protease in the apoptotic pathway among the many caspases [57]. This study confirmed that the last step in the apoptosis cascade is caspase-3 (the final executioner of the apoptosis pathway) via a change in the expression of caspase-3, confirmed by the trend of the apoptosis rate. 

Selenium is an essential dietary trace element, which is the active center of various selenoproteins and determines their physiological functions [15,58]. At least four kinds of selenoproteins are found in the endoplasmic reticulum, including selenoproteins N, T, S, and K (SEPN1, SELT, SELS, and SELK) [18,19,20,21], which play essential roles in regulating intracellular calcium concentrations or ER stress [22,23]. The expression of these four genes regulated by dietary Se has been observed in many recent studies [59,60]. Selk, which is located in ER and plasma membrane, is a newly identified selenoprotein, which may be required for normal development but does not reveal changes in the total antioxidant status of embryos and cells [61]. Furthermore, a recent study suggested that the expression of Selk may increase the secretion of insulin in the endoplasmic reticulum (ER) [62]. Selk might increase lipid consumption in the liver and then suppress NAFLD and NASH. Sepn1 has been shown to be responsible for a genetic disorder and is the first selenoprotein identifiably related to this function [63]. Sepn1 shows a calcium-binding site and a motif similar to those observed in the catalytic sites of thioredoxin reductases, another selenoprotein subgroup [64]. We, however, found no function assigned to Sepn1. The function of Selt, therefore, remains unclear (it has been considered to be a putative Sec-containing redox center in humans). Selt is similar to Sep15, SelM, and SelW, suggesting its suitability in the catalysis of redox reactions [65]. Sels is present in a variety of tissues, such as liver, skeletal muscle, and adipose tissue, and is an endoplasmic reticulum (ER) transmembrane protein [66]. This ER membrane protein is involved in stress responses to prevent the accumulation of the deleterious consequences of a misfolded protein, which has been connected to immune and inflammatory processes [67]. In this study, we found that the intervention of Se on HFD-induced NASH reached its highest point at a Se concentration of 1.0 mg/kg and then reduced as the dietary Se level increased.

On the other hand, the highest relative mRNA abundance of the Sels gene was observed in the pigs fed the BD/HFD diet containing 1 mg/kg Se. However, after reaching the maximum level, further increases in Se dose led to a reduction in Sels mRNA expression in the liver. We found an inverse correlation between the expression of Sels and the trend of the apoptosis rate and symptoms of NASH. Similar to our result, a previous study indicates that the overexpression of Sels might defend Min6 cells against oxidative stress inducer H_2_O_2_-induced cell death [68]. Kim et al. (2007) also revealed that the overexpression of Sels might defend macrophages against ER stress-induced cytotoxicity and apoptosis; conversely, in macrophages, the suppression of Sels resulted in the sensitization of cells to ER stress-induced cell death [18]. This result suggests that a Se concentration of 1.0 mg/kg may be optimum to protect the liver from NASH with an HFD and that Sels may play a vital role in this effect.

## 5. Conclusions

In conclusion, the results of the present findings indicated that the consumption of HFD can significantly elevate the activities of ALT and AST, induce oxidative damage and apoptosis in liver, which destroyed liver structure. It shows that the supplement of Se at 1.0 mg/kg may be the optimum concentration against damage induced by HFD, and Sels may play a vital role in the process. Further studies on the analysis of additional factors related to selenoproteins are needed to clarify the mechanisms of ER and apoptosis in NAFLD induced by HFD.

## Figures and Tables

**Table 1:** Ingredient and nutrient levels of the basal diets.

	BD(g/kg)		HFD(g/kg)			
Ingredients	10–30 kg	30–60 kg	60–110 kg	10–30 kg	30–60 kg	60–110 kg
Corn grain	721.59	753.64	802.36	670.88	655.14	654.9
Soybean meal (43.00% CP)	220	195	150	231	214	178
Wheat bran	20	20	20	20	20	20
Lard	0	0	0	30	70	110
Fish oil	0	0	0	10	10	10
L-lysine	7.24	4.11	3.9	6.95	3.62	3.15
DL-methionine	0.86	0.15	0.04	0.89	0.24	0.12
L-threonine	0.86	0	0	0.85	0	0
L-tryptophan	0.15	0	0.1	0.13	0	0.03
Salt	3	3	3	3	3	3
Limestone	9.8	10.6	9.6	9.8	10	9.3
Dicalcium phosphate	10.5	7.5	5	10.5	8	5.5
Premix ^1^	6	6	6	6	6	6
Calculated analysis %						
Digestible energy kcal/kg	3296	3294	3303	3492	3688	3929
Crude protein	16.1	15	13.4	16.2	15	13.4
Crude fat	3	3.1	3.2	6.8	10.7	14.6
Crude fiber	3.1	3	2.8	3	2.9	2.7
ADF *	4.14	4.04	3.82	4.08	3.90	3.61
NDF *	9.94	9.94	9.85	9.60	9.25	8.82
Calcium	0.7	0.65	0.55	0.7	0.65	0.55
Available phosphorus	0.3	0.25	0.21	0.3	0.25	0.21
Lysine	1.15	0.92	0.8	1.15	0.92	0.8
Methionine	0.34	0.26	0.23	0.34	0.26	0.23
Methionine + Cysteine	0.63	0.54	0.49	0.63	0.54	0.48
Threonine	0.68	0.56	0.5	0.68	0.56	0.5
Tryptophan	0.19	0.16	0.15	0.19	0.17	0.15

Note: ^1^ Vitamin and mineral premix (by per kilogram of diet): Gu: 10 mg; Fe: 100 mg; Mn: 4 mg; Zn: 100 mg; I: 0.2 mg; Se: 0 mg; vitamin A, 3000 IU; cholecalciferol, 300 IU; vitamin E, 20 IU; menadione, 1.0 mg; thiamin, 2 mg; riboflavin, 5 mg; pyridoxine, 12 mg; vitamin B12, 0.04 mg; pantothenic acid, 20 mg; niacin, 40 mg; folic acid, 0.6 mg; biotin 0.1 mg. * ADF, acid detergent fiber; NDF, neutral detergent fiber.

**Table 2:** Primer sets for qPCR.

Gene	Accession Number	Primer sequence 5′→3′
P53	NM_213824.3	F: GTCACGAACTGGCTGGATG
		R: GAAGGGACAAAGGACGACAG
Bax	XM_003355975.2	F: TGCTTCAGGGTTTCATCCAG
		R: GACACTCGCTCAACTTCTTGG
Bcl-2	XM_003474076.2	F: CCTTCTCCGTGGTCATCCT
		R: AAGTCTGAGCGTCCTGTTCC
Caspase-3	NM_214131.1	F: GTGGGATTGAGACGGACAG
		R: TTCGCCAGGAATAGTAACCAG
Selk	DQ372075	F: CAGGAAACCCCCCTAGAAGAA
		R: CTCATCCACCGGCCATTG
Sepn1	EF113595	F: ACCTGGTCCCTGGTGAAAGAG
		R: AGGCCAGCCAGCTTCTTGT
Sels	GU983865	F: ACAGGAGGCTTTAGCAGCAG
		R: CGCTGTCCCATCTTTCAATC
Selt	NM_001163408	F: CGCTGCTCAAATTCCAGATA
		R: CTCTCCTTCAATGCGGATGT
β-actin	AY550069	F: CCCAAAGCCAACCGTGAGAA
		R: CCACGTACATGGCTGGGGTG

**Table 3:** Results of the growth performance.

Items		Growth Performance			
Fat	Se	ADG	ADFI	Carcass Weight	Carcass Length
	(mg/kg)	(kg/d)	(kg/d)	(kg/d)	(cm)
BD	0	0.73 ± 0.01 ^c^	1.90 ± 0.21	63.66 ± 7.52	66.67 ± 2.52
	0.3	0.70 ± 0.02 ^b^	1.85 ± 0.15	61.67 ± 6.93	70.67 ± 0.58
	1	0.73 ± 0.07 ^c^	1.93 ± 0.10	62.50 ± 5.07	68.33 ± 2.08
	3	0.67 ± 0.04 ^a^	1.84 ± 0.18	59.33 ± 5.01	70.33 ± 3.61
HFD	0	0.87 ± 0.11 ^B^*	2.17 ± 0.12 *	78.83 ± 7.37 ^B^*	83.00 ± 6.25 ^B^*
	0.3	0.86 ± 0.06 ^B^*	2.14 ± 0.09*	77.33 ± 1.04 ^B^*	73.00 ± 1.00 ^A^
	1	0.82 ± 0.03 ^A^*	2.00 ± 0.11	74.33 ± 4.65 ^B^*	72.33 ± 0.58 ^A^
	3	0.83 ± 0.08 ^A^*	2.06 ± 0.10 *	72.00 ± 2.78 ^A^*	73.23 ± 5.77 ^A^
*p*-value					
Fat		<0.01	<0.01	<0.01	<0.01
Se		0.25	0.47	0.38	0.06
Fat × Se		0.27	0.28	0.91	<0.01

Note: * Different from the BD at that Se addition, *p* < 0.05. ^a–c^ Within BD groups, means without a common letter differ, *p* < 0.05. ^A–C^ Within HFD groups, means without a common letter differ, *p* < 0.05.

**Table 4:** Results of the levels of ALT and AST in the serum.

Items		Aminotransferase	
Fat	Se (mg/kg)	ALT (U/L)	AST (U/L)
BD	0	58.16 ± 11.23	129.32 ± 11.31 ^c^
	0.3	52.76 ± 5.43	98.10 ± 6.22 ^a^
	1.0	48.08 ± 5.88	88.70 ± 3.98 ^a^
	3.0	53.58 ± 9.26	115.14 ± 10.70 ^b^
HFD	0	96.48 ± 10.39 ^C^*	188.66 ± 8.35 ^C^*
	0.3	83.18 ± 7.80 ^B^*	160.90 ± 8.64 ^B^*
	1.0	70.42 ± 3.90 ^A^*	137.20 ± 7.53 ^A^*
	3.0	93.04 ± 4.28 ^C^*	169.54 ± 23.92 ^C^*
*p*-value			
Fat		<0.01	<0.01
Se		<0.01	<0.01
Fat × Se		0.07	0.01

Note: * Different from the BD at that Se addition, *p* < 0.05. ^a–c^ Within BD groups, means without a common letter differ, *p* < 0.05. ^A–C^ Within HFD groups, means without a common letter differ, *p* < 0.05.

**Table 5:** Results of the levels of antioxidative parameters.

Items		Antioxidant Index	
Fat	Se (mg/kg)	GSH-Px (U/mg protein)	MDA (nmol/mg protein)
BD	0	23.24 ± 2.66 ^a^	2.51 ± 0.41 ^bc^
	0.3	128.11 ± 5.61 ^c^	2.11 ± 0.26 ^ab^
	1.0	148.09 ± 8.20 ^d^	1.94 ± 0.26 ^a^
	3.0	100.66 ± 6.04 ^b^	2.64 ± 0.35 ^c^
HFD	0	22.71 ± 2.26 ^A^	3.96 ± 0.70 ^B^*
	0.3	85.03 ± 3.25 ^C^*	3.68 ± 0.20 ^B^*
	1.0	117.14 ± 5.19 ^D^*	2.58 ± 0.34 ^A^*
	3.0	64.14 ± 4.24 ^B^*	4.26 ± 1.10 ^B^*
*p*-value			
Fat		<0.01	<0.01
Se		<0.01	<0.01
Fat × Se		<0.01	0.16

Note:* Different from the BD at that Se addition, *p* < 0.05. ^a–d^ Within BD groups, means without a common letter differ, *p* < 0.05. ^A–D^ Within HFD groups, means without a common letter differ, *p* < 0.05.

**Table 6:** Results of the concentrations of TC and TG.

Items		Antioxidant Index	
Fat	Se (mg/kg)	TC (mmol/L)	TG (mmol/L)
BD	0	1.13 ± 0.09 ^b^	0.6 ± 0.02 ^a^
	0.3	1.21 ± 0.07 ^c^	0.68 ± 0.02 ^b^
	1.0	1.07 ± 0.04 ^a^	0.68 ± 0.02 ^b^
	3.0	1.51 ± 0.07 ^d^	0.94 ± 0.03 ^c^
HFD	0	2.07 ± 0.08 ^C^*	1.26 ± 0.03 ^C^*
	0.3	1.85 ± 0.09 ^B^*	1.08 ± 0.01 ^B^*
	1.0	1.75 ± 0.04 ^A^*	0.90 ± 0.02 ^A^*
	3.0	1.83 ± 0.05 ^B^*	1.32 ± 0.03 ^C^*
*p*-value			
Fat		<0.01	<0.01
Se		<0.01	<0.01
Fat × Se		<0.01	<0.01

Note:* Different from the BD at that Se addition, *p* < 0.05. ^a–d^ Within BD groups, means without a common letter differ, *p* < 0.05. ^A– C^ Within HFD groups, means without a common letter differ, *p* < 0.05.

## Abbreviations

ADF: acid detergent fiber; ADFI, average daily feed intake; ADG, average daily gain; ALT, alanine aminotransferase; AST, aspartate aminotransferase; BD, basal diet; Bax, Bcl2-associated X protein; Bcl2, B-cell lymphoma 2; ER, endoplasmic reticulum; GPx, glutathione peroxidase; GSSG, oxidative glutathione peroxidase; H&E, hematoxylin and eosin; HFD, high-fat diet; IR, insulin resistance; MDA malondialdehyde; NAFLD, nonalcoholic fatty liver disease; NASH, nonalcoholic steatohepatitis; NDF, neutral detergent fiber; NRC, National Research Council; Se, Selenium; SELK, selenoproteins K; SELS selenoproteins S; SELT, selenoproteins T; SEPN1, selenoproteins N; SOD, superoxide dismutase; TC, total cholesterol; TG, triglyceride; TUNEL, TdT-mediated dUTP nick-end labeling.
